# First Preliminary Report on Isolation and Characterization of Novel *Acinetobacter* spp. in Casing Soil Used for Cultivation of Button Mushroom, *Agaricus bisporus* (Lange) Imbach

**DOI:** 10.1155/2011/790285

**Published:** 2011-10-11

**Authors:** D. K. Choudhary

**Affiliations:** Department of Science, Faculty of Arts, Science & Commerce (FASC), Mody Institute of Technology & Science (MITS), Sikar, Rajasthan, Lakshmangarh 332311, India

## Abstract

Despite evaluation of large number of agroindustrial wastes for their use as casing material for *Agaricus bisporus* (Lange) Imbach cultivation, scant attention has been given to the importance of biological properties of casing materials. In the present study, an attempt was made to characterize the bacterial flora in casing layer, namely, Farm Yard Manure (FYM) and Spent Mushroom Substrate/spent compost (SMS/SC) (FYM+SC, 3 : 1) and FYM and Vermi Compost (VC) (FYM+VC, 3 : 1), employing partial 16S rDNA sequencing. Available data showed a significant variety of organisms that included *Acinetobacter* and *Pseudomonas* of the **γ**-proteobacteria, that were the most frequently encountered genera. This is the first preliminary report on the microbial diversity of casing soils and demonstrates the presence of *Acinetobacter* spp. that has not been previously described in casing material.

## 1. Introduction

 Cultivation of button mushroom, *Agaricus bisporus *(Lange) Imbach, is a biotechnological process that recycles lignino-cellulosic wastes (spent substrates), and the spent substrates can be used in different ways [1, 2]. Consistent production of successful mushroom crops is built upon scientific knowledge and practical experiences. *A. bisporus* (Lange) Imbach, is the most widely cultivated species of edible mushrooms and it is the most popular cultivar among the artificially grown fungi of the world that contributes about 31.8% to the global mushroom cultivation and 85% of the total produce in India [3]. *A. bisporus* requires two different substrates to form the fruit bodies, that is, the compost for nutrition on which it grows vegetatively and the nutrient deficient casing soil in which the suitable physicochemical/biological conditions stimulate the initiation process of pin head formation for fruit body production [2]. 

 The casing layer is one of the important growing parameters and source of variation in production, quality, and uniformity of commercial cropping. A variety of casing materials have been used worldwide, among these, use of farm yard manure (FYM), as a casing medium for mushroom cultivation, has been in vogue in Indian subcontinent because of its easy availability and nonavailability of peat moss generally used for casing in Europe and USA. The advantages of using FYM as a casing material over other agroindustrial wastes have been highlighted in recent reports [4, 5]. Spent mushroom substrate (SMS) is the remnant of compost from which mushrooms have been produced and for which several important roles have been described [6]. Despite evaluation of large number of agroindustrial wastes for their use as casing material in *A. bisporus* cultivation, scant attention has been given to the importance of biological properties of the casing layer [7]. Bacteria present in casing layer considerably influence the growth and morphogenesis of *A. bisporus *production. It supports beneficial microbial populations that release growth stimulating substances, which are reportedly involved in stimulating the initiation of pin heads. Several reports are available on the beneficial effects of casing soil microbes, especially, *Pseudomonas putida* and *Alcaligenes faecalis *on *A. bisporus* [8]. 

 Not much information is available in the literature about the role of associated microflora in the casing layer and how the resident microflora in casing layer interacts with the vegetative mycelium of *A. bisporus*. There are two production-related issues requiring attention of researchers in casing soil microbiology, namely, (i) the composition of microorganisms present in casing soil and (ii) their influence on mushroom production *in situ. *Several methods and approaches are now available to generate information on microorganisms that reside in casing layer, which allow better assessment of microbial flora, wherein molecular tools for the identification of microorganisms are now in common use, and 16S rRNA gene analysis is intensively used in phylogenetic investigations. Furthermore, 16S rDNA sequence typing approach now permits identification of the surviving and culturable bacterial species, based on employing highly conserved 16S rDNA oligonucleotide primers for the eubacteria with an intervening hypervariable gene sequence, which could be used as signature sequence to aid in species identification [9, 10]. The objective of this present study was to characterize the bacterial flora of casing layer and impact of casing on production of button mushroom.

## 2. Materials and Methods

### 2.1. Sampling Site

In the present study casing material included spent mushroom substrate/spent compost (SMS/SC) and vermicompost (VC). Both the materials were amended with farm yard manure (FYM) in the ratio of 3 : 1. These casing materials were collected from Mushroom Research and Training Centre (MRTC), GB Pant University of Agriculture & Technology, Pantnagar, India. Samples were drawn from successive stages of each casing, namely, 0-day casing (when casing was applied over the mycelium impregnated compost, that is, at the time of casing), mycelium impregnated stage (MIS) of casing, casing at primordial stage (PS), and harvesting stage (HS) casing.

### 2.2. Physicochemical and Nutrient Analysis of Casing Mixtures

Processed casings (formalin treated, 2% v/v) were used for physicochemical analysis using methods suggested by various workers: bulk density [11], water holding capacity [12], porosity [13], electrical conductivity and pH (TPS Smartchem Laboratory Analyzer), total organic matter and C [14], available N [15], available P [16], and available K [17]. For macro- and micronutrients (Ca, Mg, Cu, Zn, Mn, and Fe) analysis procedure of Tandon [18] was employed.

### 2.3. Bacterial Isolation from Casings

Casing sample (10 g) was suspended in 90 mL of 0.85% normal saline (pH 7.0) and shaken vigorously at 150 rpm at 18°C for 1 h. The resulting slurry was serially diluted (100 *μ*L) to 900 *μ*L of 0.85% normal saline in each Eppendorf tube, and appropriate dilution (10^−4^) of this suspension (100 *μ*L) was spread plated in triplicate, on King's B medium [19]. Cultures were incubated at 20°C ± 2 for 2 days. For experimental use, isolates were transferred when needed to King's B medium that was stored at 4°C.

### 2.4. Morphological Characteristics

A total of 38 isolates were randomly selected, morphologically from all the four successive stages of casing soil. Cellular morphology and biochemical characteristics were determined based upon Bergey's manual (http://www.bergeys.org/).

### 2.5. Recovery of Genomic DNA and PCR

Total DNA, from bacterial isolates, was prepared following the procedure outlined by Bazzicalupo and Fani [20]. Extracted genomic DNA was run in 0.8% agarose gel at 80 V for 45 min with quantitative marker in one lane (Low DNA Mass Ladder, MBI Fermentas). Gel was visualized under UV transilluminator. DNA was quantified spectrophotometrically, by measuring OD at 260 nm and 280 nm. Purity of DNA was checked measuring the extinction at A_260_/A_280_, on a DU 640 B Beckman spectrophotometer. The amplified 16S rRNA gene was obtained from each bacterial isolate by PCR amplification, employing the eubacterial universal primers [21]: fDI (5′-AGAGTTTGATCCTGG-3′) and rP2 (5′-TACCTTGTTACGACTT-3′), which were targeted at universally conserved regions and permitted amplification of approximately 1,500-bp fragment. PCR amplification was carried out in a PTC-100 thermocycler (M. J. Research). Reaction tubes contained 25 ng (5 *μ*L) of DNA extract, 1 U of *Taq* polymerase (Genei), 1 X buffer (10 mM Tris-Chloride [pH 9.0], 1.5 mM MgCl_2_, 500 mM KCl) (Genei), 10 mM dNTPs (Genei), and 0.25 mM of each primer (Genei). Initial DNA-denaturation and enzyme activation steps were performed at 95°C for 7 min, followed by 25 cycles of denaturation at 94°C for 1 min, annealing at 51°C for 1 min and extension at 72°C for 1 min, and a final extension at 72°C for 10 min. The presence and yield of specific PCR product (16S rRNA gene) were monitored on 0.8% agarose (wt./vol.) (Life Technologies Inc.); gel electrophoresis was carried out at 100 V for 30 min in 1 X Tris-acetate-EDTA buffer and visualization by ethidium bromide staining and viewing on a UV transilluminator (Biosystematica).

### 2.6. Partial Sequencing of the 16S rDNA

PCR products obtained from 38 bacterial strains were purified with an EXO-SAP. Components were supplemented with gold buffer (Applied Biosystem) and sequenced on an Applied Biosystem 310 Genetic analyzer (ABI Prism 310 Genetic analyzer), using big dye terminator cycle sequencing Ready Kit (Lab India). The partial sequences, amplified by the fDI primer, were used to determine the similarities. The homology analysis for bacterial strains, based on partial sequences, was compared with the sequences from the DNA databases, and similar sequences showing above 95% were retrieved by nucleotide Basic Local Alignment Search Tool (BLAST) program at the National Center for Biotechnology Information (NCBI) BLAST server (http://www.ncbi.nlm.nih.gov/BLAST). Multiple sequence alignment of retrieved sequences was done by EBI ClustalW server http://www.ebi.ac.uk/Tools/msa/clustalw2/. Homology tree was constructed by using MEGA 4.0.2 software with bootstrap values in cluster algorithm, Phylip format, and topological algorithm. The sequences determined in this study have been submitted in the GenBank database of NCBI and discussed under accession numbers: AY961042-61, AY 967718-967724, and DQ074744-53/58.

## 3. Results

### 3.1. Physicochemical Properties of Casing

The casing sample FYM+SMS (3 : 1) had minimum bulk density (BD) (0.60 g/cm^3^), while the casing FYM+VC (3 : 1) showed a BD value of 0.68 g/cm^3^. The porosity was significantly high (92.0%) for FYM+SMS. Water holding capacity appears directly related to porosity and bulk density and may directly affect microbial build up and yield of *A. bisporus*. It was high (191.19%) for FYM+SMS. A significant increase in electrical conductivity (EC) was observed for FYM+VC (570.33 deci-simen^−^), whereas low value (398.00 deci-simen^−^) was obtained for FYM+SMS. Water holding capacity appears directly related to porosity and bulk density. It was maximum (191.19%) for FYM + SMS (3 : 1) (Table 1).

### 3.2. Macro- and Micronutrients Analysis of Casing

*Agaricus bisporus* was grown on wheat-straw-based mushroom compost (pasteurized/long method). Two casing mixtures were prepared from three casing materials: Farm Yard Manure (FYM), Spent Mushroom Substrate (SMS), and Vermi Compost (VC) were evaluated for their nutritional status. In order to assess the nutritional status of casing mixtures (FYM+SMS, FYM+VC) during various stages of cropping, status of availability macro- and micronutrients were analyzed (Table 2). Maximum level of organic matter was recorded from the two casing mixtures (28.91%  ± 0.2 and 26.42%  ± 0.08) collected at mycelium-impregnated stage, followed by the pinning stage (27.76%  ± 0.2 and 25.82%  ± 0.3). Minimum organic matter for FYM+SMS and FYM+VC was recorded in samples that collected at the harvesting stage (26.06%  ± 0.08 and 25.02%  ± 0.6); 0 day stage casing soil mixtures showed comparatively lesser organic matter content, that is, 25.38%  ± 0.11 and 24.81%  ± 0.2. A significant variation was recorded in FYM+SMS casing preparation which showed different nutritional status than FYM+VC. Micronutrients estimation (ppm) included Cu, Zn, Mn, and Fe. The relative order of nutritional status (macro- and micronutrients) for various stages of casing mixture was mycelium-impregnated stage > pinning state > harvesting stage >0 day stage (Table 2).

### 3.3. Population Dynamics of Recovered Isolates

**Figure 1:**
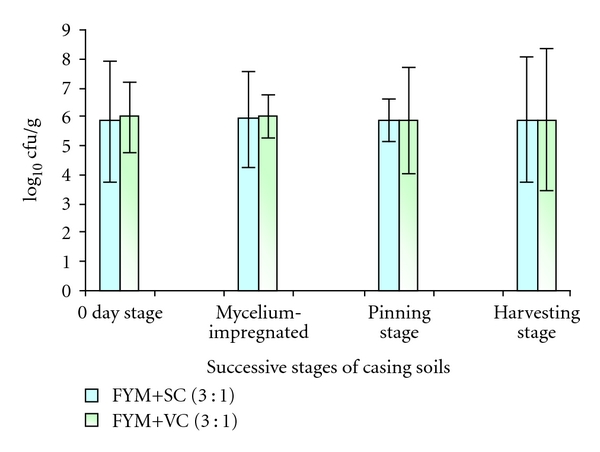
Total population count log⁡_10_⁡cfu of recovered mesophilic bacteria on KBA from successive stages of casing soils. Numerals; (1) 0-day stage, (2) mycelium-impregnated stage (MIS), (3) pinning stage (PS), (4) harvesting stage (HS).

There was little variation (6.02 ± 0.81 to 5.87 ± 0.81) in total bacterial population counts (log⁡_10_⁡CFU) among casing layers FYM+SMS and FYM+VC tried at successive stages of cropping (Figure 1). The casing layer FYM+VC from the mycelium-impregnated stage showed slightly higher population (6.02 ± 0.81) compared to FYM+SMS (5.92 ± 1.69). The lowest population was observed at pinning stage in FYM+SC (5.87 ± 0.81).

### 3.4. 16S rRNA Gene Partial Sequencing

**Figure 2:**
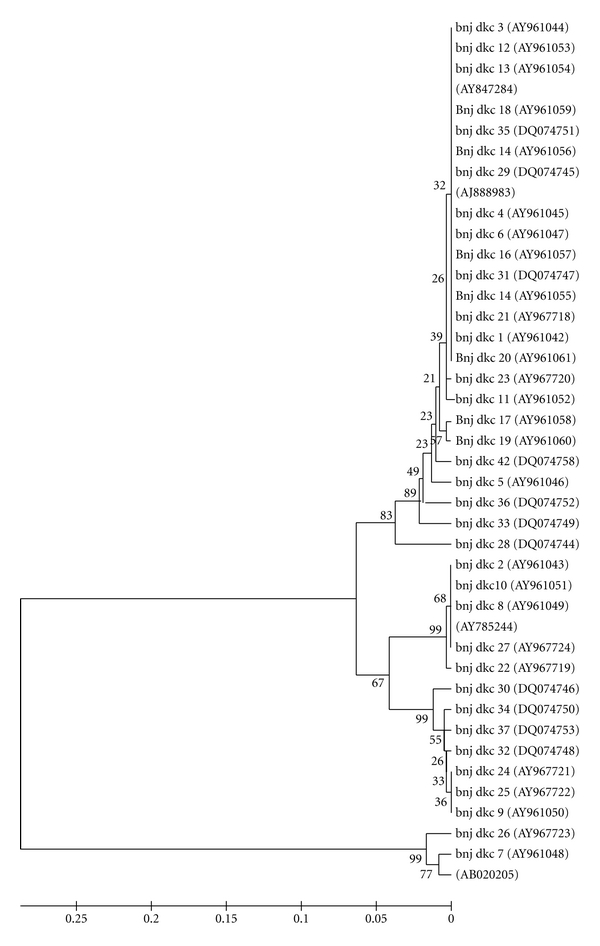
Multiple sequence alignment of retrieved sequences and homology tree constructed by using MEGA 4.0.2 software with bootstrap values for 38 bacterial isolates recovered from casing soils and their comparison with sequences (AB020205, AY847284, AJ888983, and AY785244) borrowed from the GenBank.

Partial 16S rDNA sequences were determined for 38 isolates and compared with the sequences from the DNA databases and similar sequences showing above 95% were retrieved by nucleotide Basic Local Alignment Search Tool (BLAST) program at the National Center for Biotechnology Information (NCBI) BLAST server (http://www.ncbi.nlm.nih.gov/BLAST). Most strains were grouped together and were close to the genus *Pseudomonas* and *Acinetobacter* of the family Pseudomonadaceae and Moraxellaceae, respectively, besides, others showed 55% similarity with reference strain *Sphingobacterium* (Figure 2). This bacterium (Sphingobacteriaceae family) showed Gram stain negative and exhibited NO_3_ reductase and catalase activity along with citrate utilization. A total of 11 strains (Pseudomonadaceae family), out of 38, exhibited close proximity with the genus *Pseudomonas*. All the 11 strains showed 95–99% similarity with reference strain *Pseudomonas putida* (AY785244). Large numbers of strains were distributed in family Moraxellaceae and they exhibited close proximity to genus *Acinetobacter* and showed Gram stain negative and exhibited NO_3_ reductase and catalase activity along with citrate utilization.

## 4. Discussion

Considering the significance of mushroom production in the country and, yet, limited availability of information on casing soil microflora, an exhibitive analysis of bacterial diversity characterization of this unique material was undertaken. The casing material was selected that its nutritional status was very low compared to that of compost and was thus expected to create conditions of nutritional stress. If the casing material is rich in organic matter, it is likely to disturb the selectivity of compost and thus provide continued support to the vegetative growth of the fungus and thus discourage fruiting. Characteristically, microorganisms bring about physical and chemical changes to their habitats [22]. Water holding capacity appears directly related to porosity and bulk density. These factors are directly affecting microbial build up and yield of *A. bisporus*. It was maximum (191.19%) for FYM+SMS (3 : 1). In general increase in electrical conductivity (EC) is almost proportional to decrease in number of pinheads [23]. Data of the present investigation on EC (Table 1) exhibited that the higher EC of the casing FYM+VC (3 : 1), the lower the yield. The results indicate that the EC plays an important role in the production of button mushroom, but it is not the sole controlling factor. These findings are in agreement with the finding of Bhatt et al. [24]. In addition, physicochemical properties of Indian casing materials have also been reported by Singh et al. [25]. The macro- and micronutrients level increased and decreased correspondingly (Table 2). These modifications, whether ephemeral or enduring, altered the environment so that some species were able to live and flourish, while others may have adapted and evolved to occupy the novel habitats, while still others were eliminated or failed to grow [22]. 

Molecular tools for the identification of casing soil bacteria were used and 16S rRNA gene analysis was intensively used to understand the homology relationships. Comparative analysis with BLAST sequences revealed that 85% of the bacterial isolates belonged to *γ*-proteobacteria group and other isolates were bacilli. A single isolate in this study was found to belong to the genus *Sphingobacterium.* Two genera, *Acinetobacter* and *Pseudomonas,* were dominant and were sole representative of *γ*-proteobacteria. Dominance of the genus *Acinetobacter *was of significance since this has not earlier been reported from the mushroom casing ecosystem. Presence of *Acinetobacter* as a dominant genus was not totally surprising since this bacterium is an important component of the natural ecosystem, notably involved with the degradation of lignin containing compounds and xenobiotics and its scavenging property for other noxious compounds. Based on partial 16S rRNA gene amplification and sequencing, Watabe et al. [26] examined several genera and species, including *Bacillus licheniformis*, *B. subtilis*, *Paenibacillus lentimorbus*, *Pseudomonas mevalonii*, *Sphingobacterium multivorum,* and *Stenotrophomonas *sp. in addition to two potentially novel species within the genera *Microbacterium *and *Stenotrophomonas*.

## 5. Conclusions

In this preliminary study, 16S-rDNA PCR and direct automatic sequencing of the amplicons were only carried out on culturable organisms isolated on the nonselective King's medium B (KMB). In conclusion, this is the first preliminary report on the microbial diversity of casing soils and that demonstrates the presence of *Sphingobacterium *sp. and *Acinetobacter* sp. that has not been previously described in casing material.

## Figures and Tables

**Table 1:** Physicochemical properties of casing mixtures.

S. No.	Casing soil	Properties				
		Bulk density (g/cm^3^)	Porosity (%)	Water holding Capacity (%)	pH	Electrical conductivity (deci-simen^−1^)
(1)	FYM+SC; 3 : 1	0.60	92.00	191.19	7.21	398.00
(2)	FYM+VC; 3 : 1	0.68	82.00	95.97	7.13	570.33

**Table 2:** Chemical characteristics with respect to macro- and micronutrients for casing mixtures recovered during the successive stages of button mushroom.

Characteristics	FYM+SC (3 : 1)				FYM+VC (3 : 1)			
	0 day stage	Mycelium-impregnated stage	Pinning stage	Harvesting stage	0 day stage	Mycelium-impregnated stage	Pinning stage	Harvesting stage
Organic matter (%)	25.38 ± 0.11	28.91 ± 0.20	27.76 ± 0.20	26.06 ± 0.08	24.81 ± 0.20	26.42 ± 0.08	25.82 ± 0.30	25.02 ± 0.16
*Macronutrients *								
Organic C (%)	14.10 ± 0.14	16.06 ± 0.16	15.42 ± 0.14	14.47 ± 0.23	13.78 ± 0.07	14.67 ± 0.01	14.34 ± 0.01	13.9 ± 0.2
N (%)	0.92 ± 0.02	1.41 ± 0.05	1.32 ± 0.04	1.09 ± 0.09	1.02 ± 0.04	1.33 ± 0.08	1.21 ± 0.02	1.15 ± 0.40
P (%)	0.78 ± 0.01	1.2 ± 0.09	1.05 ± 0.01	0.98 ± 0.04	0.8 ± 0.05	1.08 ± 0.02	0.94 ± 0.04	0.90 ± 0.02
K (%)	0.9 ± 0.02	1.21 ± 0.01	1.01 ± 0.02	1.06 ± 0.19	0.83 ± 0.12	1.16 ± 0.01	1.04 ± 0.06	0.94 ± 0.11
Ca (ppm)	11.51 ± 0.18	13.72 ± 0.08	13.8 ± 0.16	12.91 ± 0.07	7.99 ± 0.14	11.76 ± 0.04	10.16 ± 0.01	9.69 ± 0.16
Mg (ppm)	5.23 ± 0.02	5.01 ± 0.02	4.98 ± 0.02	4.84 ± 0.02	6.33 ± 0.01	7.89 ± 0.18	7.56 ± 0.05	5.8 ± 0.01
*Micronutrients *								
Cu (ppm)	0.025 ± 1.65	0.037 ± 1.03	0.032 ± 0.97	0.028 ± 1.04	0.016 ± 0.01	0.023 ± 0.96	0.019 ± 1.43	0.017 ± 1.36
Zn (ppm)	0.128 ± 0.04	0.416 ± 0.01	0.329 ± 0.01	0.282 ± 0.01	0.067 ± 0.11	0.269 ± 0.08	0.188 ± 0.01	0.221 ± 0.01
Mn (ppm)	1.01 ± 0.01	1.2 ± 0.02	1.16 ± 0.03	1.10 ± 0.01	0.86 ± 0.02	1.04 ± 0.16	0.98 ± 0.05	0.91 ± 0.02
Fe (ppm)	0.059 ± 0.01	0.088 ± 0.02	0.076 ± 0.03	0.063 ± 0.01	0.034 ± 0.02	0.056 ± 0.01	0.048 ± 0.01	0.041 ± 0.02
